# Low-invasive 5D visualization of mitotic progression by two-photon excitation spinning-disk confocal microscopy

**DOI:** 10.1038/s41598-021-04543-7

**Published:** 2022-01-17

**Authors:** Takafumi Kamada, Kohei Otomo, Takashi Murata, Kaito Nakata, Shota Hiruma, Ryota Uehara, Mitsuyasu Hasebe, Tomomi Nemoto

**Affiliations:** 1 Graduate School of Information Science and Technology, Hokkaido University, Sapporo, Japan; 2 Research Institute for Electronic Science, Hokkaido University, Sapporo, Japan; 3 Biophotonics Research Group, Exploratory Research Center for Life and Living Systems, Okazaki, Japan; 4 Division of Biophotonics, National Institute for Physiological Sciences, Okazaki, Japan; 5 Department of Physiological Sciences, School of Life Science, SOKENDAI, Okazaki, Japan; 6 Graduate School of Medicine, Juntendo University, Tokyo, Japan; 7 Division of Evolutionary Biology, National Institute for Basic Biology, Okazaki, Japan; 8 Department of Basic Biology, School of Life Science, SOKENDAI, Okazaki, Japan; 9 Department of Applied Bioscience, Kanagawa Institute of Technology, Atsugi, Japan; 10 Graduate School of Life Science, Hokkaido University, Sapporo, Japan; 11 Faculty of Advanced Life Science, Hokkaido University, Sapporo, Japan

**Keywords:** Multiphoton microscopy, 3-D reconstruction, Time-lapse imaging, Cellular imaging, Fluorescence imaging

## Abstract

Non-linear microscopy, such as multi-photon excitation microscopy, offers spatial localities of excitations, thereby achieving 3D cross-sectional imaging with low phototoxicity even in thick biological specimens. We had developed a multi-point scanning two-photon excitation microscopy system using a spinning-disk confocal scanning unit. However, its severe color cross-talk has precluded multi-color simultaneous imaging. Therefore, in this study, we introduced a mechanical switching system to select either of two NIR laser light pulses and an image-splitting detection system for 3- or 4-color imaging. As a proof of concept, we performed multi-color fluorescent imaging of actively dividing human HeLa cells and tobacco BY-2 cells. We found that the proposed microscopy system enabled time-lapse multi-color 3D imaging of cell divisions while avoiding photodamage. Moreover, the application of a linear unmixing method to the 5D dataset enabled the precise separation of individual intracellular components in multi-color images. We thus demonstrated the versatility of our new microscopy system in capturing the dynamic processes of cellular components that could have multitudes of application.

## Introduction

Fluorescence microscopy is an essential tool for visualizing both static structures and dynamic processes in biological samples^1,2^. One of the most commonly used fluorescence microscopies is confocal laser scanning microscopy (CLSM), which realizes cross-sectional imaging at the focal plane by adding confocal pinholes in front of the fluorescence detector^3^. Two-photon excitation laser scanning microscopy (TPLSM) also acquires such cross-sectional images because the fluorophores are excited in the focal volume of the objective lens by near-infrared (NIR) laser light pulses^4^. The NIR wavelength also offers the essential advantage of avoiding photodamage in thick biological specimens. Moreover, the fluorophores are excited throughout the optical path of an excitation light beam in CLSM, while only fluorophores at the cross-sectional focal plane are excited in TPLSM. From the viewpoint of the laser scanning mechanics, LSMs can be classified into a single-point-scanning, a multi-point-scanning, or a 2D- or 3D-structured light illumination type tool. Although most of commercially available TPLSMs have adopted single-point-scanning, several methodologies for multi-point-scanning have been proposed since the early twenty-first century^5^. Among these, we have focused on spinning-disk scanning units for achieving high-speed TPLSM imaging^6–8^. The main part of the unit is composed of a microlens-array disk and a pinhole-array (Nikpow) disk, which enables simultaneous scanning of specimens with several hundred foci. These two disks were required to be customized to increase the throughput of the excitation laser light for adopting TPLSM^9^. In addition, the Nipkow-disk provides the TPLSM system confocality, especially the axial spatial resolution is superior to that of the conventional TPLSM systems^6,9^.

Biological imaging often requires simultaneous several-color visualizations for understanding the molecular, cellular, or tissue interactions^10^. However, due to the near wavelength emission spectra of fluorophores, spectral overlaps often generate cross-talk between each color channel, which in turn disturbs distinguishing of the individual targets. In particular, a combination of fluorophores is often excited simultaneously only by a single wavelength in the two-photon excitation process. The simultaneous excitations resulting from two-photon excitation cross-section spectra of fluorophores are generally broader in shape than that from their single-photon excitation ones^11^. Recently, a linear unmixing methodology was proposed for canceling the overlapping of fluorescence from simultaneously excited fluorophores^3^. This method assumed that the total detected signals for every channel were expressed as a linear combination of the contributing fluorophores, which enabled calculations to isolate each signal of the individual fluorophore as long as some different spectral properties.

In this study, we successfully demonstrated high-speed 4-channels imaging by introducing a mechanical fast-selecting system for either of the two types of NIR laser light beams and an image-splitting detection system into our TPLSM by utilizing a spinning-disk scanner (TPLSM-SD). Moreover, the linear unmixing method developed here canceled out the cross-talks among the 4-channels images in the acquired dataset. Our developed methodology could successfully help visualizations of the 3D dynamics of individual organelles undergoing mitosis with low photodamage.

## Results

**Figure 1:**
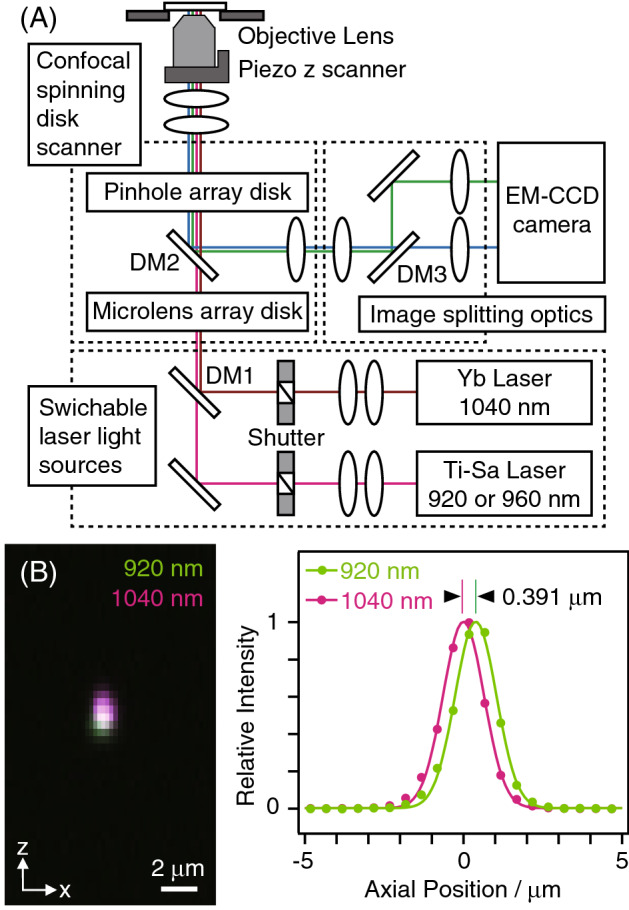
(**A**) Optical schematics of TPLM-SD with switching between two lasers and the use of image-splitting optics. (**B**) Chromatic aberration evaluation of the system between 920-nm and 1040-nm excitations. Merged 3D fluorescent images (Left panel) and axial fluorescent intensity profiles (Right panel) of a Nile Red-labeled bead of diameter 1 µm.

For fast imaging and individual visualizations of 3 major fluorescent proteins (i.e., green, yellow, and red fluorescence), a titanium-sapphire (Ti-Sa) laser light source, an ytterbium (Yb)-based laser light source, and a dichroic beam splitter equipping fluorescence image-splitting optics were applied to our TPLSM-SD system (Fig. 1A). By alternating the excitation beamlines with mechanical shutters, we achieved fast 4-channel imaging (Ch1: Ti-Sa laser light pulses excitation, shorter-wavelength emission; Ch2: Ti-Sa laser light pulses excitation, longer-wavelength emission; Ch3: Yb laser light pulses excitation, shorter-wavelength emission; Ch4: Yb laser light pulses excitation, longer-wavelength emission). Before measurements of the biological specimens, we confirmed a chromatic aberration of the system for different excitation wavelengths by evaluating the *xyz* fluorescence images of a fluorescent bead of diameter 1 µm (Fig. 1B). Comparison of the axial focal positions between 920-nm of the Ti-Sa laser and 1040-nm of the Yb laser revealed a ca. 0.4-µm difference along the optical axis, which might be negligible considering the larger 0.6–0.7-µm focal spot size of the system^6^.

**Figure 2:**
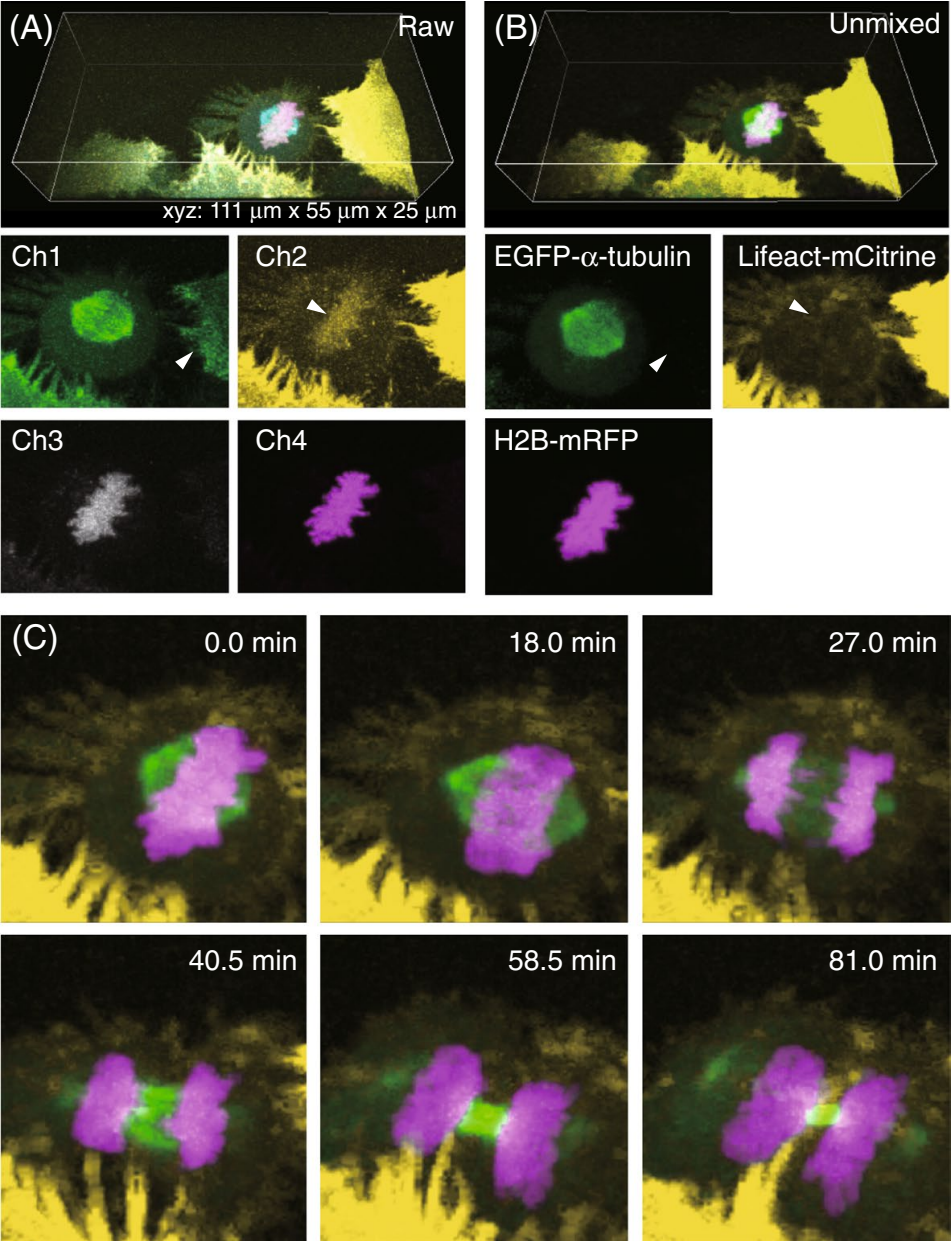
The *xyzt-λ* images of 3-color labeled human HeLa cells undergoing mitosis measured by the TPLSM-SD system. (**A**) Raw images at the time point of 0.0 min. Arrowheads represent leaking signals due to inter-channel cross-talks. (**B**) Calculated images by a linear unmixing method. Arrowheads representing the area where the leaking signals were decreased. (**C**) Time-lapse images of cell divisions during mitosis. *Z*-stacks (25-µm-thick) were taken at 0.5-µm intervals. In each sectioned image, the exposure times for 920-nm and 1040-nm excitations were 60 ms and 420 ms, respectively. The total time for *xyz-λ* image acquisition was 33 s, while the volume time-lapse interval was 90 s for 82-min measurements. The averaged laser power at the position of specimen for 920-nm and 1040-nm excitations were 162 mW and 37 mW, respectively.

As a proof of concept, we applied the developed system to conduct *xyzt*-*λ* imaging in human HeLa cells expressing Lifeact-mCitrine, EGFP-α-tubulin, and histone H2B-mRFP (labeling filamentous actin (F-actin), microtubules, and chromosomes, respectively)^12,13^. As shown in Fig. 2A, although the 4-channel fluorescent images were visualized with submicrometer resolutions, inter-channel cross-talks were recorded; the EGFP and mCitrine signals were detected in both Ch1 and Ch2, while the mRFP signals were detected in Ch2, Ch3, and Ch4. To overcome this issue, we applied a linear unmixing method based on firmly established procedure^3,14^ to the measured raw dataset. Initially, the matrix *R* was obtained from the image datasets of the HeLa cells labeled with an individual single fluorophore, which was acquired with the same condition as in the subsequent multi-color imaging (Supplementary Table S1A). After normalization to an identity matrix, the matrix was transposed to *R*^+^ (Supplementary Table S1B). By multiplying *R*^+^ with the 4-channel signal intensity matrices measured in raw images, individual fluorophore images were successfully isolated as shown in Fig. 2B. For instance, in the unmixed images, the mCitrine signals were not detectable in the EGFP-labeled microtubules image, unlike in the Ch1 image without unmixing. For quantitative evaluations, we compared the signal intensities of F-actin, which were arrowhead indicated in Fig. 2A,B, by using the signal intensities of the microtubules in the neighboring cell as the standard. As the result, the leaking signals of the F-actin were decreased by 80% after the unmixing. As for the mCitrine-labeled F-actin images, the mRFP signals were mostly undetectable, unlike in the Ch2 image. We also evaluated the signal intensities of chromosomes indicated with arrowheads in Fig. 2A,B based on the F-actin signal intensities as the standard, realizing that the unmixing decreased the leaking chromosomes signals by 96%. Next, every *xyz*-*λ* image at each time point was converted to an individual fluorophore image. Using this approach, the complex real-time morphological changes of different subcellular structures during mitosis, including the formation of the mitotic spindles, segregation of the chromosomes, and reorganization of the actin cytoskeleton, could be traced in a 3D manner (Fig. 2C, Supplementary Video S1).

Since mitotic control is susceptible to photodamage, intensive excitation light irradiations for detailed multi-dimensional measurements often cause blockage of the mitotic progression^15^. To evaluate photodamage resultant from the series of measurements using the new microscopy system, we investigated the impacts of successive imaging on the mitotic progression of HeLa cells stably expressing EGFP-α-tubulin and H2B-mRFP. For comparison, we tested the impact of successive imaging by a conventional single-photon excitation spinning-disk confocal microscopy system equipped with a single 488-nm laser light source. We selected cells that showed initiated chromosome condensation for the *xyzt*-*λ* imaging and determined whether mitosis was successful based on the presence or absence of chromosome segregation, respectively, during the subsequent 60-min imaging (Supplementary Fig. S1A). For the single-photon excitation imaging, the 488-nm excitation laser power was adjusted to obtain a similar extent of fluorescent intensities with the two-photon excitation imaging. We acquired *xyzt*-*λ* images during the mitosis of HeLa cells expressing EGFP-α-tubulin and histone H2B-mRFP with the same exposure time of the camera and spatiotemporal intervals. As the result, the progression of the mitosis was prevented in 4 out of 9 tested cells (44%) (Supplementary Fig. 1B), suggesting that the measurement condition was near the breaking point. In contrast, the two-photon excitation imaging inhibited the mitosis of HeLa cells expressing Lifeact-mCitrine, EGFP-α-tubulin, and histone H2B-mRFP in only 1 out of 10 cells (10%). These results indicate the less invasiveness of the newly developed system when compared to that of the conventional single-photon excitation.

**Figure 3:**
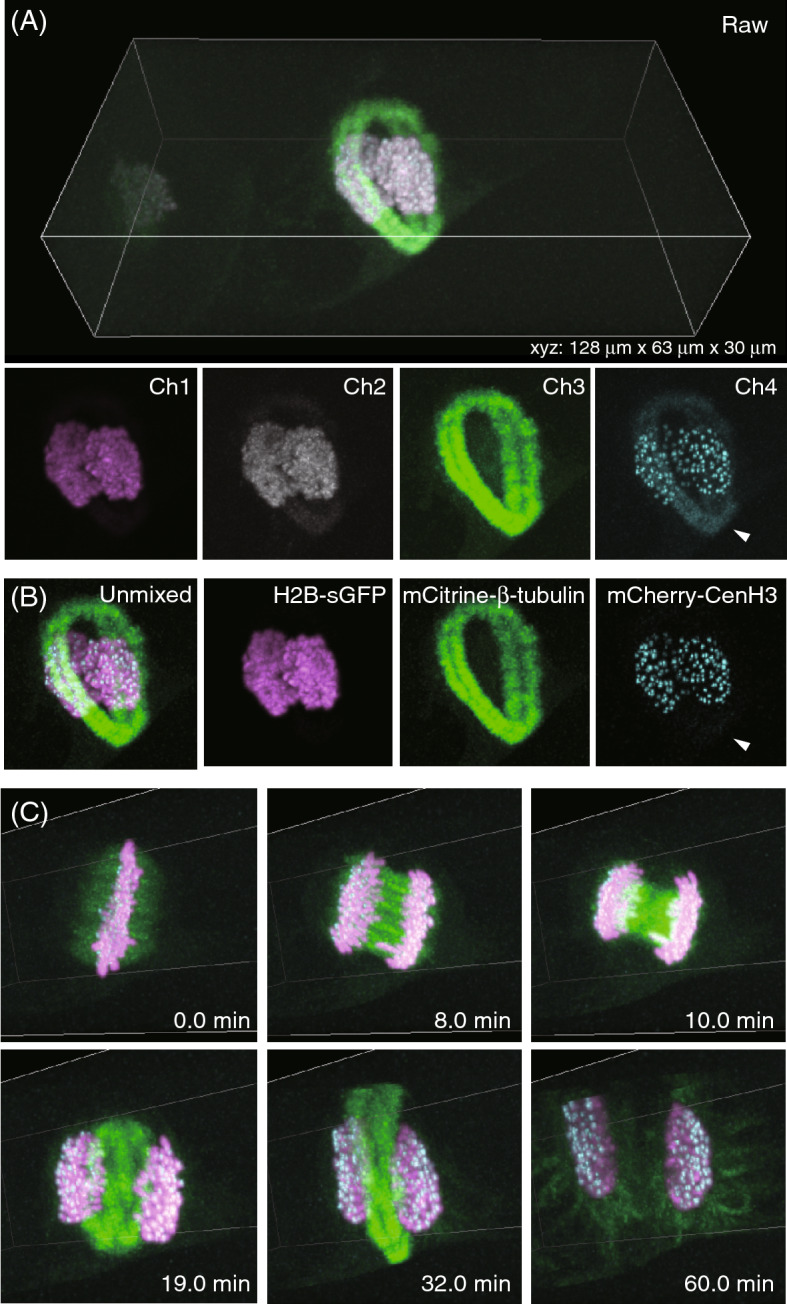
The *xyzt-λ *images of 3-color labeled tobacco BY-2 cells undergoing mitosis, as measured by the TPLSM-SD system. (**A**) Raw images at the time point of 32.0 min. Arrowheads representing leaking signals due to the inter-channel cross-talks. (**B**) Calculated images by a linear unmixing method. Arrowheads representing the area where the leaking signals were decreased. (**C**) Time-lapse images of cell divisions during mitosis. *Z*-stacks (30-µm-thick) were taken at 0.5-µm intervals. In each sectioned image, the exposure times for both 920-nm and 1040-nm excitation were 300 ms. The total time for *xyz-λ* image acquisition was 50 s, and the volume time-lapse interval was 60 s for 60-min measurements. The averaged laser power at the position of specimen for 920-nm and 1040-nm excitations were 162 mW and 37 mW, respectively.

Next, we observed multi-color labeled tobacco BY-2 cells, of size greater than the mammalian cells. Since plant cells tended to exhibit stronger light refractions and scattering than animal cells in general, intracellular structures were difficult to be visualized by single-photon excitation confocal microscopy, especially at the far side of the cell from the objective lens^16^. For 3-colors imaging, we prepared a stable transformant of BY-2 cells that expressed H2B-sGFP^17^, mCitrine-β-tubulin, and mCherry-CenH3; these were transfected into BY-2 cells to label chromosomes, microtubules, and centromeres, respectively. As shown in Fig. 3A, individual organelles were successfully visualized from the surface to the bottom of large cylindrical cells, almost isotropically. As demonstrated for HeLa cells, inter-channel color cross-talks were also recorded in the raw images. Accordingly, we applied the linear unmixing method to the *xyz*-*λ* images of 3-color labeled BY-2 cells, and calculated the *R* and *R*^+^ matrices (Supplementary Table S2). By multiplying *R*^+^ with 4-channel signal intensity matrices measured in raw images, individual fluorophore images were successfully obtained (Fig. 3B). The calculated 3 images represented clearer morphological structures of individual organelles. Especially, in the image of centromere markers, leaking signals of mCitrine-labeled microtubules were drastically decreased than those in the Ch4 image without unmixing. We evaluated the signal intensities of microtubules, which were arrowheads indicated in Fig. 3A,B, by using the signal intensities of the centromeres as the standard. As the result, the leaking microtubules signals were decreased by 73% after the unmixing. We next applied the unmixing method all *xyz*-*λ* images at different time points. Figure 3C and Supplementary Video S2 exhibit the real-time morphological changes in individual organelles undergoing mitosis in a 3D manner. With this approach, detailed 3D morphological dynamics of mitotic spindle-phragmoplast transition, expansion, and disappearance of the phragmoplast, as well as microtubule reorganization around the nuclei at the end of mitosis were successfully traced.

**Figure 4:**
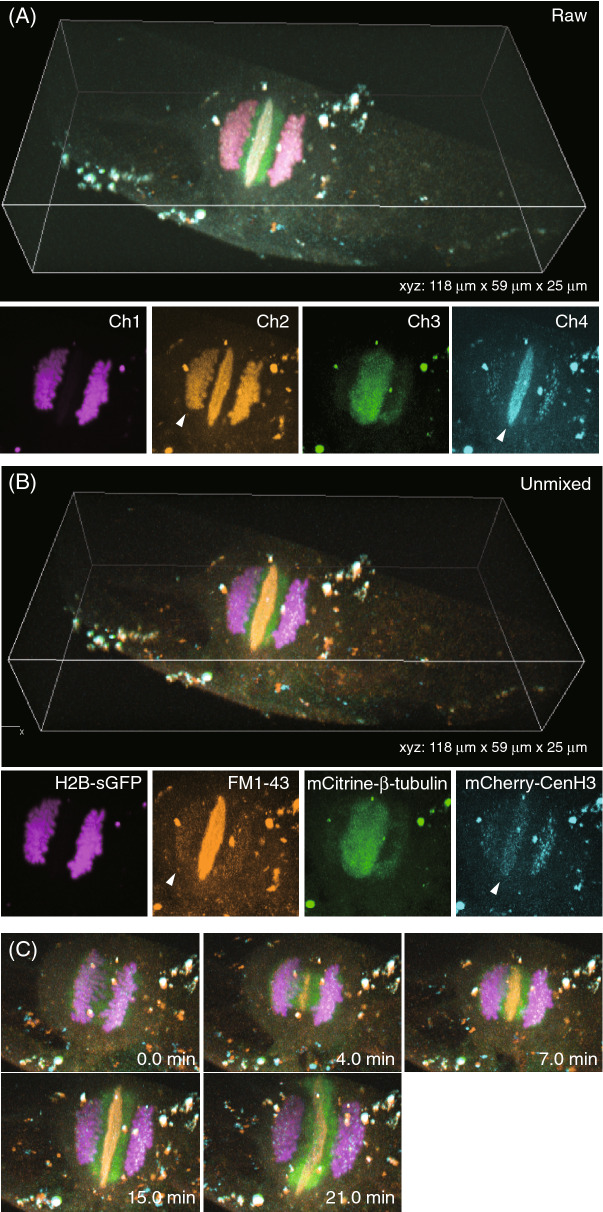
The *xyzt-λ* images of 4-color labeled tobacco BY-2 cells undergoing mitosis, as measured by the TPLSM-SD system. (**A**) Raw images at the timepoint of 15.0 min. Arrowheads representing leaking signals due to inter-channel cross-talks. (**B**) Calculated images by a linear unmixing method. Arrowheads representing the area where the leaking signals were decreased. (**C**) Time-lapse images of cell divisions during mitosis. *Z*-stacks (25-µm-thick) were taken at 0.5-µm intervals. In each sectioned image, the exposure times for both 960-nm and 1040-nm excitation were 300 ms. The total time for *xyz-λ* image acquisition was 44 s, while the volume time-lapse interval was 60 s for 23-min measurements. The averaged laser power at the position of specimen for 960-nm and 1040-nm excitations were 122 mW and 37 mW, respectively.

Finally, we demonstrated the *xyzt*-*λ* imaging of 4-color labeled specimen, by staining the above-mentioned 3-color labeled BY-2 cells using a lipophilic fluorescent dye, FM1-43, which enabled the visualization of the formation of cell plate partitioning the mother cell (Fig. 4A). However, inter-channel cross-talks were noted to be severer than in the experiments depicted in Fig. 3A. The fluorescent signals of the chromosomes and the cell plate were comparably detected in the Ch2 image. Moreover, the signals of the cell plate were approximately 32% stronger than the centromeres in the Ch4 image. Presumably because of the wider spectrum width than that of general fluorescent proteins, the FM1-43 fluorescent signals were detected in all the channels. To circumvent this problem, we applied the linear unmixing method to the *xyzt*-*λ* images of 4-color labeled BY-2 cells and then calculated an *R* matrix (Supplementary Table S3A). Since 4-channels and 4-colors resulted in a square matrix, *R* was simply inversed to *R*^+^ after the normalization to an identity matrix (Supplementary Table S3B). By multiplying *R*^+^ with 4-channel signal intensity matrices measured in raw images, individual 4 fluorophore images were obtained. As a result, the FM1-43 image represented the cell plate, plasma membranes, and non-specifically stained debris (Fig. 4B). As for the other 3 fluorescent protein-labeled organelles, leaking signals were substantially decreased than in raw images, although they were not perfect. We evaluated the signal intensities of the chromosomes and the cell plate, which were arrowheads indicated in Fig. 4A,B, by using the signal intensities of the cell plate and the centromeres in respective images as the standards. As the results, the chromosomes signals and the FM1-43 signals were decreased by 74% and 42%, respectively. As shown in Fig. 4C and Supplementary Video S3, time-lapse images represented dynamic morphological changes of 4-individual organelles under the formation of the cell plate.

## Discussions

In HeLa cells, the comparison of the mitotic progression under the conventional single-photon excitation 2-color imaging and the two-photon excitation 3-color imaging indicated that the effects of phototoxicity were smaller in the latter (Fig. 2, Supplementary Fig. S1). This difference may be attributed to the spatial locality of the two-photon excitations, which acted as milder illuminations relative to that of the single-photon excitations that caused out-of-focus absorptions. Indeed, we cannot rule out the possibility that a certain level of damage, which is insufficient to inhibit the mitotic events, was introduced by the two-photon excitation imaging. For evaluating the phototoxicity more quantitatively, the elementary processes responsible for preventing mitotic events need to be unveiled. Since several processes generated after laser illumination, like heats and reactive oxygen species, have been supposed to be candidates^15,18–20^, the multi-color imaging methodology proposed in this study may help elucidate phototoxicities through simultaneous visualization with specific fluorescent sensors and organelles^21–23^.

In this study, the frame rate or the volume rate for the acquisition was sufficiently high for the three-dimensional visualization of dynamical morphology of intracellular organelles such as chromosomes, microtubules, F-actins, centromeres, and plasma membranes. However, it was slower when compared with the recently proposed cutting-edge ultrafast microscopic technologies^24^ and for the visualization of cellular signal transductions, changing membrane potentials, or fast tiny organelle dynamics. In this sense, light-sheet microscopy has become popular because of its several merits, like the imaging speed and the less-invasiveness. In contrast, the significant merit of our TPLSM-SD system was its simple configuration compatible with an epi-fluorescence microscope. Namely, a conventional epi-fluorescence microscope can be upgraded by installing the scanner equipped with laser light sources between the microscope and the camera, which means that the identical specimens on the conventional stage of the epi-fluorescence microscope are applied. While novel single objective lens-based system^25^ or open-top stage-based system^26^ has been developed recently for light-sheet microscopy, a technique to retain the specimen remains an issue to be tackled.

A faster acquisition rate of our TPLSM-SD system would require substituting the laser beam switching shutter for a faster one with a smaller aperture or Pockels cell^27^, followed by presenting a blade-type ones with a 1-inch. aperture. On the other hand, our TPLSM-SD system took 3.3 ms for scanning the entire field of view, when the spinning speed of disks was maximum at 10,000 rpm. A recent scientific CMOS camera that achieves kHz readout speed could acquire 4 Ch images at over 100 Hz speed. In addition, a faster method for changing the *z* position is believed to be necessary. Although the piezo *z* scanner took < 10 ms for the single stepwise motion, which was negligible in this study, continuous *z* scanning instead of stepwise one could eliminate the dead times for the stepwise motion. Because the developed system involved a chromatic aberration, as shown in Fig. 1B, the combination of the sequentially switching system of excitation laser beams and the continuous *z*-positioning should require such an objective with less chromatic aberration.

We applied linear unmixing methodology for 4-channels images to cancel out inter-channel cross-talks, so that individual visualizations of green, yellow, and red fluorophores were demonstrated. While both green and yellow fluorescent protein variants tended to have sufficient brightness and photo-stabilities in particular, overlapping of their spectra has disturbed their combined usage. Our results demonstrated that simultaneous visualizations of green and yellow ones could push to enlarge the range of choices for fluorescently labeling. On the other hand, longer wavelength near 1300-nm and high-peak-power laser light sources has been reported as the excitation light source for three-photon excitation microscopy^28^. Although the repetition rate of such light sources is generally low for obtaining high-peak-power pulses, our multi-point scanning system using spinning-disks divides the light beam for several hundred ones, resulting in negligible pixel dwell times even for fast imaging^6–8^. By adding such a laser light source to the system, a larger number of signals from two-photon excited red fluorophores, three-photon excited green fluorophores, and/or sum harmonic generations can be obtained, thereby enabling increase in the number of channels and the accuracies of linear unmixing calculations.

## Materials and methods

### Optical setup

As shown in Fig. 1A, the proposed system was equipped with a Ti-Sa laser (MaiTai eHP DeepSee; Spectra-Physics, Santa Clara, CA; tunable wavelength: 690–1040 nm, averaged power: 1.7 W at 920-nm, 1.2 W at 960-nm; pulse width: 100 fs, repetition rate: 80 MHz) and an Yb-based laser light source (femtotrain; Spectra Physics; wavelength: 1040-nm, averaged power: 4 W, pulse width: 300 fs, repetition rate: 10 MHz)^6,7^. The powers of the two types of NIR light pulses were adjusted respectively by using two sets of a half-wave plate and a Glan-laser polarizer. The oscillating wavelength of the Ti-Sa laser was selected as 920-nm in case of 3-color imaging of HeLa cells and BY-2 cells. On the other hand, the wavelength was selected as 960-nm in case of 4-color imaging of BY-2 cells in order to achieve a balance for fluorescent intensities detected in individual channels. Both the beamwidths were enlarged to approximately 10 mm in diameter by applying two beam expanders comprising pairs of plano-convex lenses. These enlarged beams were then joined into a single optical path at a first dichroic mirror (DMSP1000L; Thorlabs, Newton, NJ) and introduced into a spinning-disk scanner with 100-µm-wide pinholes aligned on the Nipkow disk (CSU-MPϕ100; Yokogawa Electric, Kanazawa, Japan)^6–9^ installed on an inverted microscope (Eclipse Ti-E; Nikon, Tokyo, Japan). For HeLa cell imaging, a stage incubator (INUG2-TIZB; Tokai Hit, Fujimiya, Japan) was used at 37 °C with 5% CO_2_. The incident excitation light beam was introduced at the pupil of the water immersion objective lens (Plan Apo IR 60X, numerical aperture: 1.27, working distance: 0.17 mm; Nikon) and focused on multiple points of a specimen. Fluorescent light acquired by the objective lens was passed through the Nipkow disk, reflected by a second dichroic mirror (700–1100-nm bandpass; Yokogawa Electric), and passed through two infrared ray cut filters (FF01-770/SP-25 × 2; Semrock, Rochester, NY). Optically filtered signals were separated into a pair of fluorescent signals using a third dichroic mirror (FF580-FDi01-25 × 36; Semrock) placed in an image-splitting optical unit (W-View Gemini; Hamamatsu Photonics, Hamamatsu, Japan). Next, they were focused on an electron-multiplying CCD camera (iXon Ultra 897: 512 pixels × 512 pixels, pixel size: 16 µm × 16 µm; Andor Technology, Belfast, UK) using relay lenses with a magnification of × 1.2. Then, *z*-scans were performed with a piezo actuator (P-721; Physik Instrumente, Karlsruhe, Germany). As for single-photon excitation imaging, a spinning-disk scanner was replaced with the conventional confocal spinning-disk scanner (CSU-10; Yokogawa Electric). An excitation laser light (Sapphire LP 488; Coherent, Santa Clara, CA) was used for continuous wave oscillating at 488-nm, which was coupled with a single-mode fiber for introducing into the scanner. The NIS-Elements C software (Nikon) was used to acquire all the images.

### Image analysis

The linear unmixing method was performed for isolating individual fluorophore signals from the multi-color imaging datasets. Assuming the concentrations of the three types of fluorophores, *C*_*A*_, *C*_*B*_, and *C*_*C*_ and detecting the signals in 4-channels, *S*_1_, *S*_2_, *S*_3_, and *S*_4_, the following linear equations can be represented:

$$\left[\begin{array}{c}{S}_{1}\\ {S}_{2}\\ {S}_{3}\\ {S}_{4}\end{array}\right]= \left[\begin{array}{ccc}{R}_{A1}& {R}_{B1}& {R}_{C1}\\ {R}_{A2}& {R}_{B3}& {R}_{C2}\\ {R}_{A3}& {R}_{B3}& {R}_{C3}\\ {R}_{A4}& {R}_{B4}& {R}_{C4}\end{array}\right]\left[\begin{array}{c}{C}_{A}\\ {C}_{B}\\ {C}_{C}\end{array}\right]$$

where *R*_*X*n_ was the contribution of chromophore *X* in channel n. These values were measured by single fluorophore-labeled specimen imaging data under the same measurement condition with main multi-color imaging. Unless measurement conditions were changed, same matrices were used for calculations. To calculate concentrations of each fluorophore out, the following equation was solved:

$$\left[\begin{array}{c}{C}_{A}\\ {C}_{B}\\ {C}_{C}\end{array}\right]=\left[\begin{array}{c}{R}_{A1}^{+}\\ {R}_{B1}^{+}\\ {R}_{C1}^{+}\end{array} \begin{array}{c}{R}_{A2}^{+}\\ {R}_{B2}^{+}\\ {R}_{C2}^{+}\end{array} \begin{array}{c}{R}_{A3}^{+}\\ {R}_{B3}^{+}\\ {R}_{C3}^{+}\end{array} \begin{array}{c}{R}_{A4}^{+}\\ {R}_{B4}^{+}\\ {R}_{C4}^{+}\end{array}\right]\left[\begin{array}{c}{S}_{1}\\ {S}_{2}\\ {S}_{3}\\ {S}_{4}\end{array}\right]$$

where the *R*^+^ based matrix was transposed one from *R* after normalization to an identity matrix. In the case of 4-color imaging, *R*^+^ was simply inverted. In addition, before applying the linear unmixing method for the detected signal datasets of every pixel, noises in the raw images were removed by a spatial median filter. Image analyses were performed by using the ImageJ (US National Institution of health), R studio (R development core team), and NIS-Elements C software (Nikon). Although the macro for linear unmixing calculations was written with R studio, there are readily available open-source software such as a plug-in for ImageJ “Spectral Unmixing”^29^ which is a standard one as ours, “Learning Unsupervised Means of Spectra (LUMoS)” which enables to separate signals blindly^30^, and many others.

### Sample preparations

Fluorescently labeled beads (Nile Red, 1-µm diameter; Thermo Fisher Scientific) were diluted in water (1:3000, v/v), applied dropwise to glass coverslips, and allowed to dry. The coverslips were then mounted using a mounting medium.

The HeLa cells stably expressing EGFP-α-tubulin and H2B-mRFP^13^ were cultured in Dulbecco’s modified Eagle’s medium (DMEM, Wako Pure Chemical, Osaka, Japan), supplemented with 10% fetal bovine serum (FBS) and 1× antibiotic–antimycotic (Sigma-Aldrich, St-Louis, MO) at 37 °C with 5% CO_2_. The cells were seeded on a glass-bottom dish for observations. The plasmid encoding Lifeact-mCitrine was constructed in our laboratory, as previously described^6^. The Lifeact-mCitrine plasmid was transiently transfected using JetPEI (Polyplus-transfection, Illkirch, France) according to the manufacturer’s instruction. After incubation for 1 day, the culture medium was switched to DMEM without phenol red (Wako Pure Chemical), containing 10% FBS and antibiotics.

The plasmids harboring H2B-sGFP, mCitrine-β-tubulin, and mCherry-CenH3 for the stable transformation of tobacco BY-2 cells were constructed as described below. The H2B coding region of tobacco genomic DNA was fused with sGFP^17^ coding sequence with a monomeric A206K mutation. The fusion gene was transfected with a custom-made binary vector, pTKM-BSD, using a blastocidin S-resistant marker gene. The vector was prepared from pCAMBIA1300 by changing the hygromycin B-resistant gene with a blastocidin S-resistant gene. For mCitrin-β-tubulin, cDNA of *Arabidopsis thaliana TUB6* (gifted from Dr. Y. Oda) was fused with a mCitrine sequence and transformed with a pRI201 binary plasmid (Takara Bio, Kusatsu, Japan) with a kanamycin-resistant gene. The 35S promoter of pRI201 was replaced with the ubiquitin promoter of *A. thaliana*. For mCherry-CenH3, the tobacco CenH3 cDNA was cloned, fused with a mCherry sequence, and transformed with pCAMBIA1300 using a Nos promoter and an *A. thaliana* heat-shock protein terminator. Agrobacterium-mediated transformation of the tobacco BY-2 cells was performed as described elsewhere^31^. The transformed cells were subcultured every week in a modified LS medium^32^. The cells were attached to a glass-bottom dish, as previously described^33^.

## Supplementary Information

Supplementary Information.

Supplementary Video 1 (avi).

Supplementary Video 1(mov).

Supplementary Video 2 (avi).

Supplementary Video 2 (mov).

Supplementary Video 3 (avi).

Supplementary Video 3 (mov).

## Data Availability

The datasets generated during and/or analysed during the current study are available from the corresponding authors on reasonable request.
