# Supplementary material for: Low-invasive 5D visualization of mitotic progression by two-photon excitation spinning-disk confocal microscopy
Source: Sci Rep. 2022 Jan 17;12:809. doi: 10.1038/s41598-021-04543-7 (PMC8764092; doi:10.1038/s41598-021-04543-7)
Supplement: Supplementary file 1 — Supplementary Information. [file 41598_2021_4543_MOESM1_ESM.pdf]

Supplementary materials for

Low-invasive 5D visualization of mitotic progression by two-photon excitation spinning-disk confocal microscopy

Takafumi Kamada, Kohei Otomo*, Takashi Murata, Kaito Nakata, Shota Hiruma, Ryota Uehara, Mitsuyasu Hasebe, Tomomi Nemoto*

*** Corresponding authors:**
Kohei Otomo, otomo@nips.ac.jp,
Tomomi Nemoto, tn@nips.ac.jp

Supplementary Figure

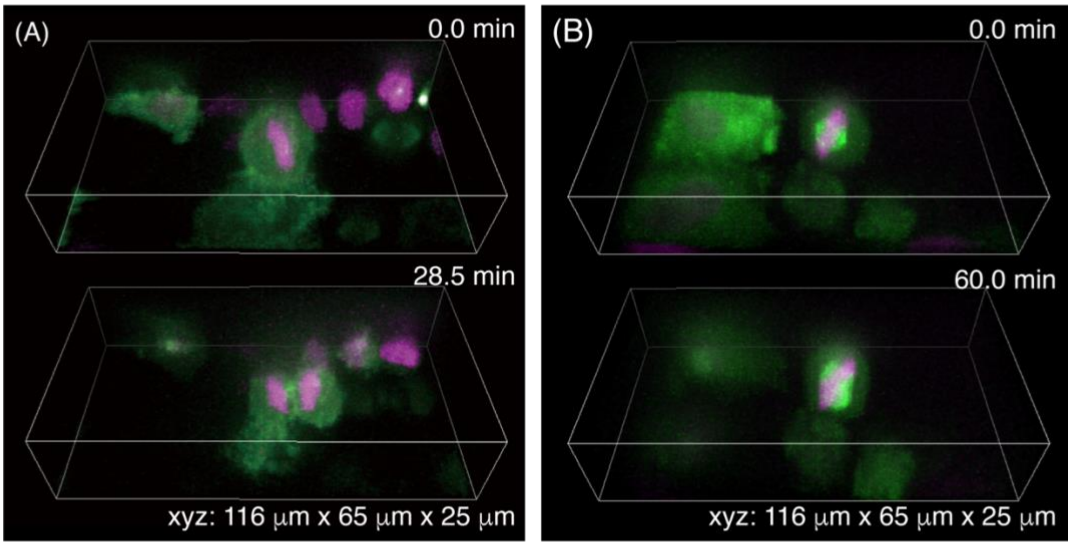

Fig. S1. The *xyzt-λ* images of 2-color labeled human HeLa cells undergoing mitosis, as measured by the single-photon excitation LSM-SD system. (A) Images of the succeeded chromosome segregation. (B) Images of the failed chromosome segregation. Time-lapse images of cell divisions during mitosis. *Z*-stacks (25-μm-thick) were taken at 0.5-μm intervals. In each sectioned image, the exposure time for 488-nm excitation was 480 ms. The total time for *xyz-λ* image acquisition was 33 s, while volume time-lapse interval was 90 s for 60-min measurements. The averaged laser power at the position of specimen for 488-nm excitations was 13 μW.

Supplementary Tables

Table S1. Values in the matrices were used for linear unmixing methods for raw imaging dataset of the 3-color human HeLa cells. (A) The row and column of the R matrix correspond to fluorescent intensities detected in each channel and the types of fluorophores, respectively. (B) R^+ matrix was transposed from the normalized identity matrix of the R matrix.

(A)

	R_{An}	R_{Bn}	R_{Cn}
R_{X1}	2468	13677	119
R_{X2}	336	2501	1653
R_{X3}	139	6520	222
R_{X4}	163	1318	4024

(B)

	R^+_{X1}	R^+_{X2}	R^+_{X3}	R^+_{X4}
R^+_{An}	7.4357	−0.2115	−15.6634	0.7730
R^+_{Bn}	−0.1640	0.1318	2.8212	−0.2053
R^+_{Cn}	−0.3933	1.3417	−0.3997	3.5109

Table S2. Values in the matrices were used for linear-unmixing methods for raw imaging dataset of the 3-color tobacco BY-2 cells. (A) The row and column of the *R* matrix correspond to the fluorescent intensities detected in each channel and the type of fluorophores, respectively. (B) R^+ matrix was transposed from the normalized identity matrix of the *R* matrix.

(A)

	R_{An}	R_{Bn}	R_{Cn}
R_{X1}	1889	110	81
R_{X2}	191	33	55
R_{X3}	40	159	88
R_{X4}	16	38	196

(B)

	R^+_{X1}	R^+_{X2}	R^+_{X3}	R^+_{X4}
R^+_{An}	1.0329	0.0105	−0.6895	−0.1216
R^+_{Bn}	−0.2451	0.0736	13.7484	−6.0834
R^+_{Cn}	−0.2154	1.8430	−2.8050	10.6706

Table S3. Values in the matrices were used for linear-unmixing methods for raw imaging dataset of the 4-color tobacco BY-2 cells. (A) The row and column of the R matrix correspond to the fluorescent intensities detected in each channel and the type of fluorophores, respectively. (B) R^+ matrix was inverted from the normalized identity matrix of the R matrix.

(A)

	R_{An}	R_{Bn}	R_{Cn}	R_{Dn}
R_{X1}	2158	71	122	3
R_{X2}	215	108	26	46
R_{X3}	38	17	168	16
R_{X4}	5	50	32	455

(B)

	R^+_{X1}	R^+_{X2}	R^+_{X3}	R^+_{X4}
R^+_{An}	1.1113	−0.6520	−0.7197	0.0832
R^+_{Bn}	−2.3058	23.4566	−1.5614	−2.2944
R^+_{Cn}	−0.0362	−2.0261	13.7090	−0.2672
R^+_{Dn}	0.2461	−2.4435	−0.7958	5.1748
